# Tetra­aqua­bis(orotato-κ*O*)cobalt(II) dihydrate

**DOI:** 10.1107/S1600536810015837

**Published:** 2010-05-08

**Authors:** Ahmet Nedim Ay, Dursun Ali Köse, Barış Tercan, Fatma Yüksel, Tuncer Hökelek

**Affiliations:** a Department of Chemistry, Hacettepe University, 06800 Beytepe, Ankara, Turkey; b Department of Chemistry, Hitit University, 19030 Ulukavak, Çorum, Turkey; c Department of Physics, Karabük University, 78050 Karabük, Turkey; d Department of Chemistry, Gebze High Technology Institute, 41400 Gebze, Kocaeli, Turkey; e Department of Physics, Hacettepe University, 06800 Beytepe, Ankara, Turkey

## Abstract

In the title Co^II^ complex, [Co(C_5_H_3_N_2_O_4_)_2_(H_2_O)_4_]·2H_2_O, the Co^II^ ion is located on an inversion center and is coordinated by two orotate (2,6-dioxo-1,2,3,6-tetrahydropyrimidine-4-carboxylate) anions and four water mol­ecules in a slightly distorted octa­hedral geometry. The dihedral angle between the carboxyl­ate group and the attached orotate ring is 1.2 (3)°. In the crystal structure, inter­molecular O—H⋯O, N—H⋯O and C—H⋯O hydrogen bonds link the mol­ecules into a three-dimensional network. π–π contacts between the orotate rings [centroid–centroid distances = 3.439 (2) and 3.438 (2) Å] further stabilize the structure.

## Related literature

For orotic acid, see: Doody *et al.* (1996 ▶); Köse *et al.* (2008 ▶); Levine *et al.* (1974 ▶); Nelson & Michael (2000 ▶); Smith & Baker (1959 ▶). For applications of metal–orotate complexes and their derivatives, see: Schmidbaur *et al.* (1990 ▶); Castan *et al.* (1990 ▶); Köse *et al.* (2006 ▶). For related structures, see: Ha *et al.* (1999 ▶); Icbudak *et al.* (2003 ▶); Karipides & Thomas (1986 ▶); Kumberger *et al.* (1991 ▶); Mutikainen (1987 ▶); Mutikainen *et al.* (1996 ▶); Nepveu *et al.* (1995 ▶); Platter *et al.* (2002 ▶); Sabat *et al.* (1980 ▶); Solbakk (1971 ▶); Sun *et al.* (2002 ▶).
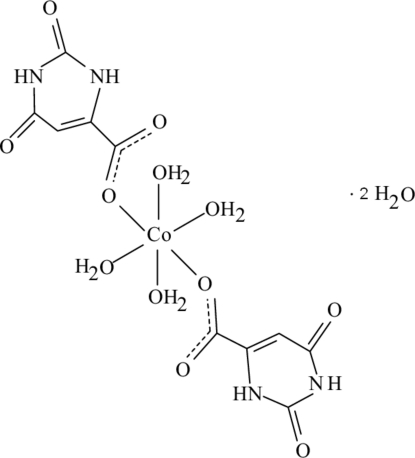


## Experimental

### 

#### Crystal data

[Co(C_5_H_3_N_2_O_4_)_2_(H_2_O)_4_]·2H_2_O
*M* *_r_* = 477.21
Monoclinic, 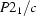

*a* = 9.8715 (5) Å
*b* = 13.1514 (7) Å
*c* = 6.7281 (3) Å
β = 92.224 (3)°
*V* = 872.81 (8) Å^3^
*Z* = 2
Mo *K*α radiation
μ = 1.07 mm^−1^
*T* = 100 K
0.35 × 0.20 × 0.15 mm

#### Data collection

Bruker Kappa APEXII CCD area-detector diffractometer
Absorption correction: multi-scan (*SADABS*; Bruker, 2005 ▶) *T*_min_ = 0.775, *T*_max_ = 0.851
6413 measured reflections
2006 independent reflections
1905 reflections with *I* > 2σ(*I*)
*R*_int_ = 0.024

#### Refinement

*R*[*F* ^2^ > 2σ(*F* ^2^)] = 0.056
*wR*(*F* ^2^) = 0.168
*S* = 1.11
2006 reflections
164 parameters
11 restraints
H atoms treated by a mixture of independent and constrained refinement
Δρ_max_ = 1.99 e Å^−3^
Δρ_min_ = −0.49 e Å^−3^

### 

Data collection: *APEX2* (Bruker, 2007 ▶); cell refinement: *SAINT* (Bruker, 2007 ▶); data reduction: *SAINT*; program(s) used to solve structure: *SHELXS97* (Sheldrick, 2008 ▶); program(s) used to refine structure: *SHELXL97* (Sheldrick, 2008 ▶); molecular graphics: *Mercury* (Macrae *et al.*, 2006 ▶); software used to prepare material for publication: *WinGX* (Farrugia, 1999 ▶) and *PLATON* (Spek, 2009 ▶).

## Supplementary Material

Crystal structure: contains datablocks I, global. DOI: 10.1107/S1600536810015837/xu2754sup1.cif

Structure factors: contains datablocks I. DOI: 10.1107/S1600536810015837/xu2754Isup2.hkl

Additional supplementary materials:  crystallographic information; 3D view; checkCIF report

## Figures and Tables

**Table 1:** Selected bond lengths (Å)

Co1—O1	2.056 (2)
Co1—O5	2.113 (3)
Co1—O6	2.115 (3)

**Table 2:** Hydrogen-bond geometry (Å, °)

*D*—H⋯*A*	*D*—H	H⋯*A*	*D*⋯*A*	*D*—H⋯*A*
N1—H1⋯O7	0.83 (4)	2.26 (4)	3.073 (4)	167 (5)
N2—H2⋯O2	0.84 (6)	1.98 (6)	2.790 (4)	162 (7)
O5—H51⋯O2	0.93 (2)	2.05 (5)	2.805 (4)	137 (5)
O5—H52⋯O7	0.91 (5)	1.82 (5)	2.710 (4)	167 (6)
O6—H61⋯O4^i^	0.95 (3)	1.84 (4)	2.781 (4)	173 (5)
O6—H62⋯O3^ii^	0.92 (5)	1.84 (5)	2.737 (4)	164 (4)
O7—H71⋯O5	0.94 (5)	1.90 (5)	2.808 (4)	160 (6)
O7—H72⋯O1	0.97 (5)	2.11 (5)	2.957 (4)	145 (6)
O7—H72⋯O6	0.97 (5)	2.44 (7)	3.201 (4)	135 (6)
C5—H5⋯O3	0.93	2.38	3.292 (5)	165

Symmetry codes: (i) 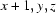; (ii) 
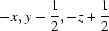
.

**Fig. 1.**
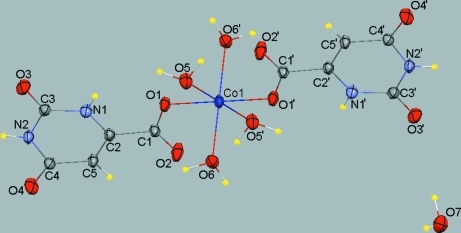
The molecular structure of the title molecule with the atom-numbering scheme. Displacement ellipsoids are drawn at the 40% probability level. Primed atoms are generated by the symmetry operator:(') -x, -y, -z.

## supplementary crystallographic information

### Comment

Orotic acid (6-uracilic acid, vitamin B13, H_3_Or) is an essential vitamin in the syntheses of pyrimidine bases of nucleic acids, since it is the first pyridine product of an enzymatic step in normal blood cells (Nelson & Michael, 2000; Smith & Baker, 1959; Levine *et al.*, 1974). Metal orotate complexes and their derivatives not only found applications in curing syndromes but also they have encouraging studies as therapeutic agents for cancer (Schmidbaur *et al.*, 1990; Castan *et al.*, 1990; Köse *et al.*, 2006). Orotic acid is an interesting ligand because it has multiple coordination sites at low and neutral pH, it is coordinated from the carboxylic acid group monodentately in ketonic form but at higher pH values bidentate coordination occurs in enolic form, the tautomerism between the ketonic and enolic structures makes multiform coordinations possible (Doody *et al.*, 1996; Köse *et al.*, 2008). Mononuclear crystal structures of Co, Cu, Mg, Ni and Zn complexes were reported, where bidentate orotate dianions (HOr^2-^) found in the molecules (Mutikainen, 1987; Mutikainen *et al.*, 1996; Icbudak *et al.*, 2003; Sabat *et al.*, 1980; Karipides & Thomas, 1986; Platter *et al.*, 2002; Kumberger *et al.*, 1991). Dianionic form of the acid (HOr^2-^) can also act as a polydentate ligand in its polymeric complexes with Cu, Ni and Mn metals (Nepveu *et al.*, 1995; Ha *et al.*, 1999; Platter *et al.*, 2002; Sun *et al.*, 2002). Relatively limited number of monoanionic orotate complex crystal studies are found in the literature. The metal orotate structures including Mg, Ni and Zn have (H_2_Or^-^) ions located in the outer coordination sphere and the monoanion does not enter the inner coordination sphere of the aquated metal, M(II), cations (Solbakk, 1971). The title compound was synthesized and its crystal structure is reported herein.

The title complex, (I), is a crystallographically centrosymmetric mononuclear complex, consisting of two orotate, (Or), ligands, four coordinated and one uncoordinated water molecules (Fig. 1). Or ligands are monodentate. The four O atoms (O5, O6, and the symmetry-related atoms, O5', O6') in the equatorial plane around the Co atom form a slightly distorted square-planar arrangement, while the slightly distorted octahedral coordination is completed by the two O atoms of the Or ligands (O1, O1') in the axial positions (Fig. 1).

The near equality of the C1—O1 [1.269 (4) Å] and C1—O2 [1.224 (5) Å] bonds in the carboxylate group indicates a delocalized bonding arrangement, rather than localized single and double bonds. The average Co—O bond length is 2.095 (3) Å (Table 1), and the Co atom is displaced out of the least-squares plane of the carboxylate group (O1/C1/O2) by 0.6042 (1) Å. The dihedral angle between the planar carboxylate group and the Or ring A (N1/N2/C2—C5) is 1.15 (31)°. Atoms O1, O2, O3, O4 and C1 are -0.064 (3), -0.027 (4), -0.069 (3), 0.040 (3) and -0.039 (4) Å away from the plane of the Or ring, respectively.

In the crystal structure, intramolecular O—H···O and intermolecular O—H···O, N—H···O and C—H···O hydrogen bonds (Table 2) link the molecules into a three-dimensional network, in which they may be effective in the stabilization of the structure. The π–π contacts between the Or rings, Cg1—Cg1^i^ and Cg1—Cg1^ii^ [symmetry codes: (i) x, 1/2 - y, z - 1/2; (ii) x, 1/2 - y, z + 1/2, where Cg1 is the centroid of the ring (N1/N2/C2—C5)] may further stabilize the structure, with centroid-centroid distances of 3.439 (2) and 3.438 (2) Å, respectively.

### Experimental

The title compound was prepared by the reaction of NH_4_H_2_Or (0.96 g, 5 mmol) in H_2_O (100 ml) and nicotinamide (1.22 g, 10 mmol) in H_2_O (100 ml) with Co(NO_3_)_2_.6H_2_O (1.45 g, 5 mmol) in H_2_O (50 ml). The mixture was filtered and set aside to crystallize at ambient temperature for one week, giving pink single crystals.

### Refinement

The highest peak and deepest hole in the final difference electron-density map were located 1.99 and 0.49 Å, respectively, from atom Co1. Atom H5 was positioned geometrically with C—H = 0.93 Å, for aromatic H atom and constrained to ride on its parent atom, with U_iso_(H) = 1.2U_eq_(C). Atoms H1, H2 (for NH), H51, H52, H61, H62, H71, H72 (for H_2_O) were located in difference Fourier maps and refined isotropically, with restrains of N1—H1 = 0.83 (2), N2—H2 = 0.84 (6), O5—H51 = 0.93 (2), O5—H52 = 0.91 (5), O6—H61 = 0.95 (2), O6—H62 = 0.92 (5), O7—H71 = 0.94 (5), O7—H72 = 0.97 (5) Å and H51-O5-H52 = 107 (4), H61-O6-H62 = 107 (4) and H71-O7-H72 = 107 (4)°.

### Crystal data

**Table:**

[Co(C_5_H_3_N_2_O_4_)_2_(H_2_O)_4_]·2H_2_O	*F*(000) = 490
*M**_r_* = 477.21	*D*_x_ = 1.816 Mg m^−^^3^
Monoclinic, *P*2_1_/*c*	Mo *K*α radiation, λ = 0.71073 Å
Hall symbol: -P 2ybc	Cell parameters from 2967 reflections
*a* = 9.8715 (5) Å	θ = 2.2–24.3°
*b* = 13.1514 (7) Å	µ = 1.07 mm^−^^1^
*c* = 6.7281 (3) Å	*T* = 100 K
β = 92.224 (3)°	Block, pink
*V* = 872.81 (8) Å^3^	0.35 × 0.20 × 0.15 mm
*Z* = 2	

### Data collection

**Table:**

Bruker Kappa APEXII CCD area-detector diffractometer	2006 independent reflections
Radiation source: fine-focus sealed tube	1905 reflections with *I* > 2σ(*I*)
graphite	*R*_int_ = 0.024
φ and ω scans	θ_max_ = 27.7°, θ_min_ = 2.1°
Absorption correction: multi-scan (*SADABS*; Bruker, 2005)	*h* = −12→11
*T*_min_ = 0.775, *T*_max_ = 0.851	*k* = −17→12
6413 measured reflections	*l* = −8→8

### Refinement

**Table:**

Refinement on *F*^2^	Primary atom site location: structure-invariant direct methods
Least-squares matrix: full	Secondary atom site location: difference Fourier map
*R*[*F*^2^ > 2σ(*F*^2^)] = 0.056	Hydrogen site location: inferred from neighbouring sites
*wR*(*F*^2^) = 0.168	H atoms treated by a mixture of independent and constrained refinement
*S* = 1.11	*w* = 1/[σ^2^(*F*_o_^2^) + (0.106*P*)^2^ + 1.3319*P*] where *P* = (*F*_o_^2^ + 2*F*_c_^2^)/3
2006 reflections	(Δ/σ)_max_ < 0.001
164 parameters	Δρ_max_ = 1.99 e Å^−^^3^
11 restraints	Δρ_min_ = −0.49 e Å^−^^3^

### Special details

**Table:**

Geometry. All esds (except the esd in the dihedral angle between two l.s. planes) are estimated using the full covariance matrix. The cell esds are taken into account individually in the estimation of esds in distances, angles and torsion angles; correlations between esds in cell parameters are only used when they are defined by crystal symmetry. An approximate (isotropic) treatment of cell esds is used for estimating esds involving l.s. planes.
Refinement. Refinement of *F*^2^ against ALL reflections. The weighted *R*-factor wR and goodness of fit *S* are based on *F*^2^, conventional *R*-factors *R* are based on *F*, with *F* set to zero for negative *F*^2^. The threshold expression of *F*^2^ > σ(*F*^2^) is used only for calculating *R*-factors(gt) etc. and is not relevant to the choice of reflections for refinement. *R*-factors based on *F*^2^ are statistically about twice as large as those based on *F*, and *R*- factors based on ALL data will be even larger.

### Fractional atomic coordinates and isotropic or equivalent isotropic displacement parameters (Å^2^)

**Table:**

	*x*	*y*	*z*	*U*_iso_*/*U*_eq_	
Co1	0.0000	0.0000	0.0000	0.0219 (3)	
O1	−0.1500 (2)	0.0836 (2)	0.1269 (4)	0.0294 (6)	
O2	−0.3106 (3)	−0.0336 (2)	0.1649 (5)	0.0388 (7)	
O3	−0.3502 (3)	0.4086 (2)	0.1788 (5)	0.0387 (7)	
O4	−0.7136 (3)	0.2040 (2)	0.2605 (5)	0.0388 (7)	
O5	−0.0640 (3)	−0.1370 (2)	0.1312 (4)	0.0310 (6)	
H51	−0.158 (2)	−0.137 (5)	0.127 (9)	0.067*	
H52	−0.038 (7)	−0.200 (3)	0.092 (12)	0.09 (3)*	
O6	0.1274 (3)	0.0294 (2)	0.2535 (4)	0.0319 (6)	
H61	0.175 (5)	0.092 (2)	0.259 (8)	0.060 (18)*	
H62	0.191 (5)	−0.021 (3)	0.278 (9)	0.057 (18)*	
O7	0.0502 (4)	0.6880 (2)	0.0068 (5)	0.0399 (7)	
H71	0.010 (7)	0.656 (5)	−0.106 (6)	0.08 (2)*	
H72	0.047 (8)	0.639 (4)	0.114 (7)	0.09 (3)*	
N1	−0.3185 (3)	0.2368 (2)	0.1577 (5)	0.0251 (6)	
H1	−0.240 (3)	0.250 (5)	0.127 (9)	0.053 (16)*	
N2	−0.5297 (3)	0.3038 (2)	0.2173 (5)	0.0269 (6)	
H2	−0.573 (7)	0.358 (4)	0.229 (12)	0.09 (3)*	
C1	−0.2701 (3)	0.0542 (3)	0.1553 (5)	0.0224 (7)	
C2	−0.3695 (3)	0.1405 (3)	0.1770 (5)	0.0216 (6)	
C3	−0.3958 (3)	0.3223 (3)	0.1841 (5)	0.0248 (7)	
C4	−0.5914 (3)	0.2099 (3)	0.2312 (5)	0.0243 (7)	
C5	−0.5017 (3)	0.1247 (3)	0.2120 (5)	0.0226 (7)	
H5	−0.5345	0.0588	0.2236	0.027*	

### Atomic displacement parameters (Å^2^)

**Table:**

	*U*^11^	*U*^22^	*U*^33^	*U*^12^	*U*^13^	*U*^23^
Co1	0.0145 (4)	0.0152 (4)	0.0363 (4)	0.00237 (19)	0.0044 (3)	−0.0016 (2)
O1	0.0167 (11)	0.0196 (12)	0.0525 (16)	0.0007 (9)	0.0096 (10)	−0.0062 (10)
O2	0.0304 (15)	0.0164 (14)	0.071 (2)	−0.0017 (11)	0.0170 (13)	0.0004 (13)
O3	0.0296 (14)	0.0192 (13)	0.0672 (19)	−0.0064 (11)	0.0013 (12)	−0.0018 (12)
O4	0.0164 (12)	0.0321 (15)	0.068 (2)	−0.0008 (10)	0.0099 (12)	−0.0026 (13)
O5	0.0220 (12)	0.0229 (13)	0.0488 (16)	0.0000 (10)	0.0085 (10)	0.0044 (11)
O6	0.0227 (13)	0.0267 (14)	0.0460 (15)	0.0023 (11)	−0.0015 (10)	−0.0024 (12)
O7	0.0453 (18)	0.0282 (15)	0.0462 (17)	0.0016 (13)	0.0007 (13)	0.0015 (12)
N1	0.0180 (13)	0.0189 (15)	0.0389 (16)	−0.0018 (11)	0.0071 (11)	−0.0014 (11)
N2	0.0189 (14)	0.0178 (14)	0.0441 (17)	0.0032 (11)	0.0045 (11)	−0.0049 (12)
C1	0.0147 (14)	0.0176 (15)	0.0351 (16)	0.0007 (12)	0.0038 (11)	−0.0011 (12)
C2	0.0180 (14)	0.0195 (16)	0.0276 (15)	0.0025 (12)	0.0042 (11)	−0.0022 (12)
C3	0.0203 (16)	0.0181 (16)	0.0359 (17)	−0.0002 (12)	0.0001 (12)	−0.0027 (12)
C4	0.0157 (14)	0.0205 (16)	0.0369 (17)	−0.0008 (12)	0.0046 (12)	−0.0051 (13)
C5	0.0165 (14)	0.0159 (15)	0.0358 (17)	0.0006 (11)	0.0055 (12)	0.0000 (12)

### Geometric parameters (Å, °)

**Table:**

Co1—O1	2.056 (2)	O6—H62	0.92 (5)
Co1—O1^i^	2.056 (2)	O7—H71	0.94 (5)
Co1—O5	2.113 (3)	O7—H72	0.97 (5)
Co1—O5^i^	2.113 (3)	N1—C2	1.371 (4)
Co1—O6	2.115 (3)	N1—C3	1.374 (5)
Co1—O6^i^	2.115 (3)	N1—H1	0.83 (2)
O1—C1	1.269 (4)	N2—C3	1.371 (4)
O2—C1	1.224 (5)	N2—C4	1.382 (4)
O3—C3	1.222 (5)	N2—H2	0.84 (6)
O4—C4	1.232 (4)	C2—C1	1.510 (4)
O5—H51	0.93 (2)	C2—C5	1.351 (4)
O5—H52	0.91 (5)	C5—C4	1.437 (5)
O6—H61	0.95 (2)	C5—H5	0.9300
			
O1—Co1—O1^i^	180.00 (14)	H71—O7—H72	107 (4)
O1—Co1—O5	92.89 (10)	C2—N1—C3	122.5 (3)
O1^i^—Co1—O5	87.11 (10)	C2—N1—H1	124 (4)
O1—Co1—O5^i^	87.11 (10)	C3—N1—H1	113 (4)
O1^i^—Co1—O5^i^	92.89 (10)	C3—N2—C4	126.8 (3)
O1—Co1—O6	89.00 (11)	C3—N2—H2	112 (6)
O1^i^—Co1—O6	91.00 (11)	C4—N2—H2	121 (6)
O1—Co1—O6^i^	91.00 (11)	O1—C1—C2	113.6 (3)
O1^i^—Co1—O6^i^	89.00 (11)	O2—C1—O1	127.2 (3)
O5^i^—Co1—O5	180.00 (19)	O2—C1—C2	119.2 (3)
O5—Co1—O6	89.84 (11)	N1—C2—C1	116.3 (3)
O5^i^—Co1—O6	90.16 (11)	C5—C2—N1	121.3 (3)
O5—Co1—O6^i^	90.16 (11)	C5—C2—C1	122.4 (3)
O5^i^—Co1—O6^i^	89.84 (11)	O3—C3—N1	123.4 (3)
O6^i^—Co1—O6	180.00 (10)	O3—C3—N2	121.8 (3)
C1—O1—Co1	126.3 (2)	N2—C3—N1	114.8 (3)
Co1—O5—H51	108 (4)	O4—C4—N2	120.2 (3)
Co1—O5—H52	124 (5)	O4—C4—C5	125.2 (3)
H52—O5—H51	107 (4)	N2—C4—C5	114.6 (3)
Co1—O6—H61	118 (3)	C2—C5—C4	119.9 (3)
Co1—O6—H62	113 (4)	C2—C5—H5	120.0
H61—O6—H62	107 (4)	C4—C5—H5	120.0
			
O5—Co1—O1—C1	−36.3 (3)	C4—N2—C3—N1	1.7 (5)
O5^i^—Co1—O1—C1	143.7 (3)	C3—N2—C4—O4	−179.6 (4)
O6—Co1—O1—C1	−126.1 (3)	C3—N2—C4—C5	1.3 (5)
O6^i^—Co1—O1—C1	53.9 (3)	N1—C2—C1—O1	1.8 (5)
Co1—O1—C1—O2	21.4 (5)	N1—C2—C1—O2	−177.8 (3)
Co1—O1—C1—C2	−158.2 (2)	C5—C2—C1—O1	−178.7 (3)
C3—N1—C2—C1	−176.5 (3)	C5—C2—C1—O2	1.8 (5)
C3—N1—C2—C5	3.9 (5)	N1—C2—C5—C4	−0.5 (5)
C2—N1—C3—O3	175.6 (4)	C1—C2—C5—C4	180.0 (3)
C2—N1—C3—N2	−4.4 (5)	C2—C5—C4—O4	179.0 (4)
C4—N2—C3—O3	−178.3 (4)	C2—C5—C4—N2	−1.9 (5)

Symmetry codes: (i) −*x*, −*y*, −*z*.

### Hydrogen-bond geometry (Å, °)

**Table:**

*D*—H···*A*	*D*—H	H···*A*	*D*···*A*	*D*—H···*A*
N1—H1···O7^ii^	0.83 (4)	2.26 (4)	3.073 (4)	167 (5)
N2—H2···O2^iii^	0.84 (6)	1.98 (6)	2.790 (4)	162 (7)
O5—H51···O2	0.93 (2)	2.05 (5)	2.805 (4)	137 (5)
O5—H52···O7^iv^	0.91 (5)	1.82 (5)	2.710 (4)	167 (6)
O6—H61···O4^v^	0.95 (3)	1.84 (4)	2.781 (4)	173 (5)
O6—H62···O3^vi^	0.92 (5)	1.84 (5)	2.737 (4)	164 (4)
O7—H71···O5^vii^	0.94 (5)	1.90 (5)	2.808 (4)	160 (6)
O7—H72···O1^viii^	0.97 (5)	2.11 (5)	2.957 (4)	145 (6)
O7—H72···O6^viii^	0.97 (5)	2.44 (7)	3.201 (4)	135 (6)
C5—H5···O3^ix^	0.93	2.38	3.292 (5)	165

Symmetry codes: (ii) ; (iii) ; (iv) ; (v) *x*+1, *y*, *z*; (vi) −*x*, *y*−1/2, −*z*+1/2; (vii) ; (viii) ; (ix) .
